# Falls Among Older Adults With and Without Alzheimer’s Disease and Related Dementias in Los Angeles County: 2016-2022

**DOI:** 10.1093/geroni/igaf122.2630

**Published:** 2025-12-31

**Authors:** D’Artagnan Robinson, Noel Barragan, Emiley Chang, Dalia Regos-Stewart, Mariana Reyes, Tony Kuo

**Affiliations:** Los Angeles County Department of Public Health, Los Angeles, California, United States; Los Angeles County Department of Public Health, Los Angeles, California, United States; Los Angeles County Department of Public Health, Los Angeles, California, United States; Los Angeles County Department of Public Health, Los Angeles, California, United States; Los Angeles County Department of Public Health, Los Angeles, California, United States; County of Los Angeles, Los Angeles, California, United States

## Abstract

Falls are a leading cause of injury, hospitalization, and mortality for older adults in the United States. Those living with Alzheimer’s disease and related dementias (ADRD) are at heightened risk for falls due to cognitive impairment and functional limitations. As part of the Los Angeles County BOLD Initiative, the Los Angeles County (LAC) Department of Public Health conducts surveillance and research on ADRD and their associated risks. Using patient discharge data from the California Department of Health Care Access and Information, this study examines fall-related hospitalizations among LAC adults aged ≥50 years during the period 2016-2022. Falls were identified using external cause of morbidity codes (ICD-10-CM: W00-W19) and ADRD status was determined via principal and secondary diagnosis codes. All falls were analyzed by admission year, age, sex, race/ethnicity and fall type (e.g., falls from same level, falls from bed), with low-frequency categories collapsed into “Other Falls.” Chi-square tests were employed to compare fall type by ADRD status. A total of n = 243,136 fall-related hospitalizations occurred during this timeframe, with 20.5% involving ADRD patients. A higher proportion of ADRD patients experienced falls from beds (8.0% vs. 4.3%, p < 0.0001) and non-motorized mobility devices (2.4% vs. 1.2%, p < 0.0001), whereas a lower proportion fell from stairs and steps (1.9% vs. 4.5%, p < 0.0001) compared to non-ADRD patients. Study findings highlight the burden of falls among this population and underscore the need to effectively prevent and manage falls, especially through strategies that address environmental and mobility risks.

